# Mitral Subvalvular Aneurysm in a Patient with Chagas Disease and Recurrent Episodes of Ventricular Tachycardia

**DOI:** 10.1155/2015/213104

**Published:** 2015-11-08

**Authors:** Tereza Augusta Grillo, Guilherme Rafael S. Athayde, Ana Flávia L. Belfort, Reynaldo C. Miranda, Andrea Z. Beaton, Bruno R. Nascimento

**Affiliations:** ^1^Electrophysiology Department, Hospital Universitário São José-INCOR Minas, 30140-073 Belo Horizonte, MG, Brazil; ^2^Interventional Cardiology Department, Hospital Universitário São José-INCOR Minas, 30140-073 Belo Horizonte, MG, Brazil; ^3^Division of Cardiology and Cardiovascular Surgery, Hospital das Clínicas, Universidade Federal de Minas Gerais, 30130-100 Belo Horizonte, MG, Brazil; ^4^Electrophysiology Department, Universidade Federal de Minas Gerais, 30130-100 Belo Horizonte, MG, Brazil; ^5^School of Medicine, Universidade Federal de Minas Gerais, 30130-100 Belo Horizonte, MG, Brazil; ^6^Children's National Health System, Washington, DC 20010, USA; ^7^Interventional Cardiology Department, Hospital das Clínicas, Universidade Federal de Minas Gerais, 30130-100 Belo Horizonte, MG, Brazil

## Abstract

Subvalvular left ventricular aneurysm is a rare disease of obscure origin suggesting unique causes such as congenital, traumatic, and inflammatory or infectious diseases. Its mortality is closely related to heart failure, mitral insufficiency, thromboembolic phenomena, and cardiac arrhythmias. Although association with coronary artery disease is not described, the compression of epicardial vessels by the aneurysm may lead to ischemic manifestations. We report here a case of mitral subvalvular left ventricular aneurysm of probable chagasic origin, in a patient with normal left ventricular function evolving with repeated episodes of monomorphic ventricular tachycardia, despite noninducible electrophysiological testing and the use of optimal medical treatment, including amiodarone. The indication for implantable cardioverter-defibrillator in patients with Chagas cardiomyopathy and segmental wall motion abnormalities but without global systolic dysfunction remains unclear in literature, even in the presence of complex ventricular arrhythmias. A brief review of the literature on morphological features, diagnosis, prognosis, and treatment will be also discussed.

## 1. Introduction

Mitral subvalvular left ventricular (LV) aneurysm is a rare abnormality of unclear, likely varied, etiology. Suggested origins include congenital defects, traumatic injuries, and the sequel of inflammatory or infectious diseases [1, 2]. Similarly, the clinical manifestations, which include heart failure, mitral regurgitation, thromboembolic phenomena, and cardiac arrhythmias, are incompletely described in literature. Sudden death is among the rare reported presentations of subvalvular LV aneurysms (both submitral and subaortic), believed to be caused by direct coronary artery compression by the aneurysm. Here, we present a case of mitral subvalvular LV aneurysm in a patient with positive Chagas serology and normal global systolic function evolving with repeated episodes of monomorphic ventricular tachycardia (MVT), refractory to oral amiodarone, despite no inducible complex arrhythmias in electrophysiological (EP) testing.

## 2. Case Report

**Figure 1:**
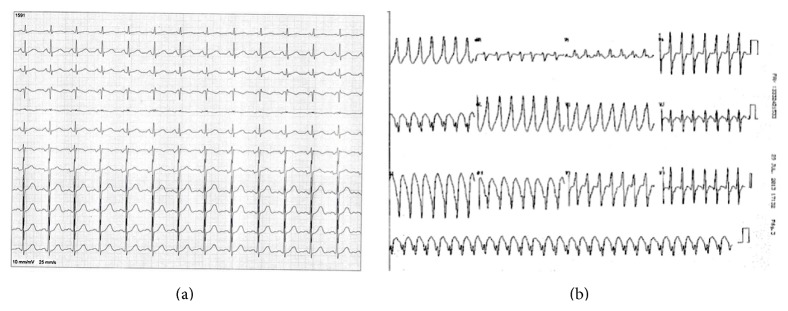
(a) Baseline electrocardiogram, showing no atrioventricular or intraventricular conduction abnormalities, nor fragmenting of the QRS complex. (b) Electrocardiogram performed in the emergency department showing monomorphic ventricular tachycardia with Rr' pattern in V1, late R/S transition, and left axis deviation.

A 42-year-old Brazilian woman presented in 2010 to the local emergency department (ED) with new-onset sustained palpitations, hemodynamically stable and denying chest pain, or dizziness. Her past medical history was negative for hospitalizations, trauma, major illness, or surgery. She took no medications and had no known drug allergies. Electrocardiogram (ECG) revealed MVT, HR = 280 bpm, with Rr' pattern in V1, late R/S transition, and left axis deviation, suggesting probable origin in the basal posterior inferior segment of the LV (Figure 1). The patient received high-dose intravenous (IV) amiodarone (300 mg), which ultimately converted the MVT to normal sinus rhythm (NSR). The baseline ECG (Figure 1) had neither atrioventricular or intraventricular conduction abnormalities, nor other abnormalities such as pathologic Q waves, low voltage, or QRS fragmentation. She was discharged on oral amiodarone 600 mg/day. In 2011 the drug was discontinued and the patient was switched to Atenolol 50 mg once daily. The patient was being managed by the primary care physician and had not received cardiology referral.

The patient remained asymptomatic in NSR until April 2012, when once more she was admitted to the ED with palpitations, weakness, dizziness, and presyncopal symptoms. On physical examination the patient was diaphoretic, hypotensive (BP 100/60 mmHg), and tachycardic (HR = 250 bpm). Again, ECG was consistent with MVT with the same pattern as the index event, which again converted to NSR with IV amiodarone.

**Figure 2:**
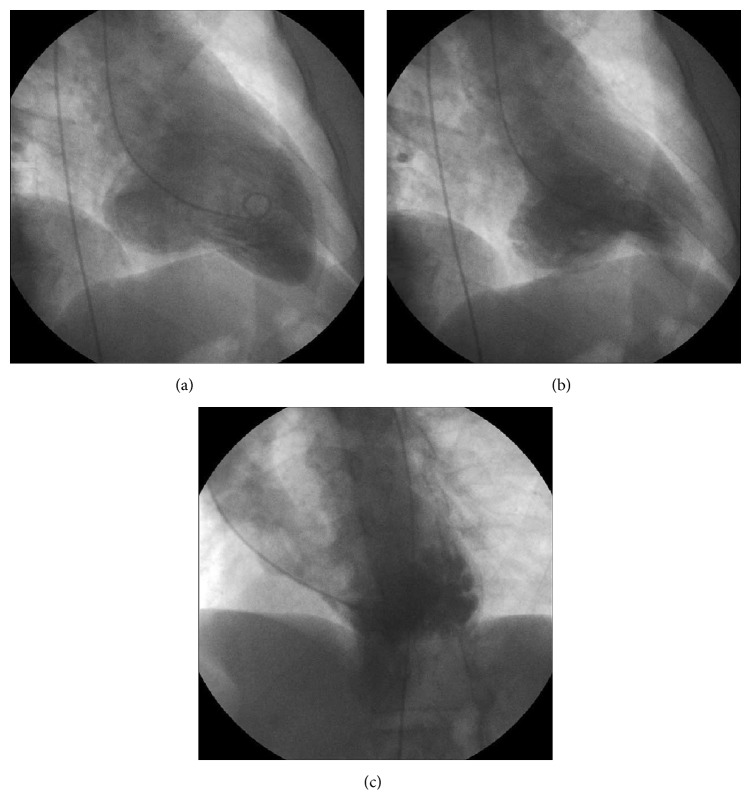
Left ventriculography, showing the mitral subvalvular aneurysm: (a) right anterior oblique, end-diastole; (b) right anterior oblique, end-systole; (c) left anterior oblique, end-systole.

Chest radiography was normal. Transthoracic echocardiography (TTE) revealed a subvalvular LV aneurysm (basal portion of the inferolateral wall) with preserved LV systolic function (LVEF = 65%), as confirmed later by ventriculography (Figure 2, Supplementary File in Supplementary Material available online at http://dx.doi.org/10.1155/2015/213104). Treadmill stress test was negative for inducible ischemia. Invasive coronary angiography was also normal (Supplementary File). Laboratory tests were positive for Chagas disease (hemagglutination inhibition and indirect immunofluorescence). Electrophysiology testing failed to induce sustained ventricular tachycardia after extensive endocardial and epicardial mapping: the programmed ventricular stimulation protocol was conducted in two basic cycles (450 and 600 ms) at the right ventricular apex and outflow tract with up to three extra stimuli and finalized when the ventricular refractory period or a minimum coupling of 200 ms was reached. As no sustained ventricular tachycardia was induced, the protocol was then repeated after infusion of Isoproterenol. No evidence of epicardial circuit was found. The noninducibility was taken as a reassurance of benign prognosis (high negative predictive value). At that point, electroanatomic mapping (EAM) was not available in our institution. She did not meet criteria for implantable cardioverter-defibrillator (ICD)—in the attending heart-team's point of view—and was discharged from the hospital after one week on oral amiodarone (800 mg/day, to be progressively reduced).

Despite good compliance with oral amiodarone, the patient once again presented to our hospital in August 2013, this time with hemodynamically unstable MVT. She was successfully converted to NSR with synchronized cardioversion. Her echocardiogram remained unchanged, and no other abnormalities were observed besides the mitral subvalvular LV aneurysm. The patient was then referred to cardiology, where the decision was made to go forward with ICD implantation. The patient remains stable and asymptomatic on oral Propafenone, with preserved LV function. She has not had recurrence of her MVT and has never required any type of therapy by her ICD after 2 years of device implantation.

## 3. Discussion

The present case illustrates a common dilemma on whether a patient with Chagas cardiomyopathy without global systolic dysfunction but with segmental wall-motion abnormalities should maintain drug therapy or have ICD implanted after an episode of sustained ventricular tachycardia without hemodynamic compromise. It also remarks the pattern of symptoms and short-term evolution of subvalvular LV aneurysm evolving with repeated episodes of MVT in which the final approach was ICD implantation.

Chagas disease affects approximately 20 million people in Latin America. Cardiac involvement is the most serious manifestation and accounts for more than 21,000 deaths every year. The majority of these deaths result from heart failure or sudden cardiac death secondary to malignant ventricular arrhythmias [3].

Our case was also complicated by the presence of a mitral subvalvular LV aneurysm, a rare cardiac abnormality first described in 1962 by Abrahams et al., thought to be caused by a congenital defect in the posterior portion of the mitral annulus and also by trauma, inflammatory, and infectious diseases, and may produce symptoms through diastolic overload [2, 4, 5], which has also been suggested as an additional focus of arrhythmogenic substratum in patients with Chagas disease [2, 4]. The mechanism of these arrhythmias is not always clear, but in some cases, compression of the circumflex artery has been observed [1, 5]. In the current case, the role of the subvalvular aneurysm is unclear, though we presume it could have contributed to the refractoriness of her ventricular arrhythmia. Indeed, while rare—Deshpande et al. reported 16 cases (0.7%) of subvalvular aneurysms from 2,285 consecutive autopsies in 10 years [1]—subvalvular aneurysms have been previously reported in patients with Chagas disease, sometimes associated with complex arrhythmias [2, 5]. In these cases, the probable arrhythmogenic substrate may also include reentry in a scar derived from necrosis and fibrosis caused by chronic inflammation of the myocardium. The reentrant circuits can originate from subendocardial, subepicardial, and intramyocardial injuries. For the latter, intramural circuits may be kept by subepicardial fibers [6].

The effectiveness of ICD for primary prevention of ventricular fibrillation (VF) and sustained VT for patients with LVEF <35%, as well as for secondary prevention of VT/VF, is well established [7] but primarily derived from data on ischemic heart disease. More recently the ACCF/AHA/HRS joint working group advocated for inclusion of other high-risk profile diseases, including Chagas cardiomyopathy, as class IIa indications for ICD implantation. This recommendation is made without regard to ventricular function, given the high risk of ventricular tachyarrhythmias in patients with Chagas [3]. Moreover, for this case, these newer 2013 recommendations also put forward a IIa-C recommendation for ICD in patients with sustained VT, regardless of hemodynamic significance [7]. Thus, our patient met two class II-a, level of evidence C recommendations.

However, this case still presented a challenge in management, as the patient presented initially with signs of good prognosis: no significant abnormalities (conduction abnormalities, pathologic Q waves, and low-voltage) in baseline ECG, preserved LV systolic function with no significant structural cardiac disease, noninducible VT on electrophysiologic study, and a good response to class III antiarrhythmic medication. The positive Chagas serology was only known after the second episode. Indeed, even if the Chagas serology had been known, there is some debate if ICD implantation prevents mortality in this population. Though the total proportion of patients with Chagas disease was low, a retrospective case review of patients with ventricular tachycardia and normal LV function showed increased all-cause mortality in patients treated with ICD plus antiarrhythmic drugs versus amiodarone alone [8]. Specifically looking at a population with Chagas disease, Leite et al. showed that, in a group of 150 patients with symptomatic VT and moderate LV dysfunction, inducible hemodynamically unstable VT was associated with higher mortality than both inducible hemodynamically stable VT and noninducible VT [9]. Contrasting this, data from patients that received ICD for secondary prevention show that Chagas disease doubles the risk of appropriate therapies and appropriate therapies or death [10]. The sample, however, included mostly patients with severe LV dysfunction, with functional impairment. In a Latin American registry that included 89 chagasic patients with ICD (91% for secondary prevention), 66% of the individuals with LV dysfunction received therapy, while only 18% of those with markers of good prognosis, such as our patient, required any therapy. Moreover, compared to our case the population was older (59 ± 10 years), 72% were male, the mean LVEF was lower (40 ± 11%), and most of the patients were symptomatic [11].

Some doubts remain regarding substrate ablation in Chagas heart disease. It is known that the disease leads to slowly progressive but incessant myocarditis, with widespread destruction of myocytes, diffuse fibrosis, mononuclear cell infiltration of the myocardium, and scarring of the conduction system [12, 13]. Thus, although the recurrence rate of malignant ventricular arrhythmias seems to decrease with the advent of EAM to establish the scar extension and limits [4, 6], it is not uncommon for patients with good LV function and favorable ablations to return 5 years afterwards presenting LV dysfunction and recurrence of new sustained ventricular tachycardias [6]. Recurrence rates are as high as 50% in some series [14], and current guidelines recommend ablation (class I) only for incessant MVT or recurrent MVT after ICD implantation [14]. In our case, it was not considered at first due to the noninducibility during endocardial and epicardial stimulation protocols. After recurrences, EAM could have been useful [4], but it was not available in our institution and in most of the tertiary hospitals in Brazil, since the Public Health System does not reimburse it. However, irrespective of availability, EAM should not defer ICD implantation [6]. There is no robust data to support surgical ventriculectomy in chagasic aneurysms [14], although there are some reports of successful suppression of ventricular arrhythmias after surgical repair of subvalvular aneurysms [15].

Given this case, we recommend routine testing for Chagas in patients presenting with VT of unclear etiology who reside in or have traveled to endemic areas. And we await more specific recommendations for indication of ICD in the context of primary prevention of sudden death in patients with Chagas disease that will be answered by the on-going CHAGASICS trial [16]. Though the data is still minimal, in the future we would consider the presence of a subvalvular aneurysm in a patient with Chagas disease an additional risk factor for malignant ventricular arrhythmias—even in the absence of global LV dysfunction—and consider this increased risk in our decision-making regarding ICD placement.

## Supplementary Material

Coronary angiogram showing no significant coronary artery disease, and left ventriculography showing the mitral subvalvular aneurysm ,in right anterior oblique and left anterior oblique views.

## References

[1] Deshpande J., Vaideeswar P., Sivaraman A. (2000). Subvalvular left ventricular aneurysms. *Cardiovascular Pathology*.

[2] García Hernández N., Espinosa Caleti B., Palacios Macedo X. (1995). Mitral subvalvular aneurysm of probable chagasic etiology. *Archivos del Instituto de Cardiologia de Mexico*.

[3] Nunes M. C. P., Dones W., Morillo C. A., Encina J. J., Ribeiro A. L. (2013). Chagas disease: an overview of clinical and epidemiological aspects. *Journal of the American College of Cardiology*.

[4] Valdigem B. P., Pereira F. B. F. C. G., Carneiro Da Silva N. J. (2011). Ablation of ventricular tachycardia in chronic chagasic cardiomyopathy with giant basal aneurysm carto sound, CT, and MRI merge. *Circulation: Arrhythmia and Electrophysiology*.

[5] Deshpande A. V., Vaidya S. V., Kumar A. (2004). Submitral aneurysm. *Heart*.

[6] Scanavacca M. (2014). Epicardial ablation for ventricular tachycardia in chronic Chagas heart disease. *Arquivos Brasileiros de Cardiologia*.

[7] Epstein A. E., DiMarco J. P., Ellenbogen K. A. (2012). 2012 ACCF/AHA/HRS focused update incorporated into the ACCF/AHA/HRS 2008 guidelines for device-based therapy of cardiac rhythm abnormalities: a report of the American College of Cardiology Foundation/American Heart Association task force on practice guidelines and the Heart Rhythm Society. *Journal of the American College of Cardiology*.

[8] Connolly S. J., Hallstrom A. P., Cappato R. (2000). Meta-analysis of the implantable cardioverter defibrillator secondary prevention trials. AVID, CASH and CIDS studies. Antiarrhythmics vs implantable defibrillator study. Cardiac arrest study Hamburg. Implantable defibrillator study. *European Heart Journal*.

[9] Leite L. R., Fenelon G., Simoes A., Silva G. G., Friedman P. A., de Paola A. A. V. (2003). Clinical usefulness of electrophysiologic testing in patients with ventricular tachycardia and chronic chagasic cardiomyopathy treated with amiodarone or sotalol. *Journal of Cardiovascular Electrophysiology*.

[10] Barbosa M. P., da Costa Rocha M. O., de Oliveira A. B., Lombardi F., Ribeiro AL. (2013). Efficacy and safety of implantable cardioverter-defibrillators in patients with Chagas disease. *Europace*.

[11] Muratore C. A., Batista Sa L. A., Chiale P. A. (2009). Implantable cardioverter defibrillators and Chagas' disease: results of the ICD registry Latin America. *Europace*.

[12] Rassi A., Rassi A., Marin-Neto J. A. (2010). Chagas disease. *The Lancet*.

[13] Ribeiro A. L., Nunes M. P., Teixeira M. M., Rocha M. O. C. (2012). Diagnosis and management of Chagas disease and cardiomyopathy. *Nature Reviews Cardiology*.

[14] Andrade J. P., Marin Neto J. A., Paola A. A. (2011). I Latin American guidelines for the diagnosis and treatment of Chagas cardiomyopathy. *Arquivos Brasileiros de Cardiologia*.

[15] Geukens R., Van de Werf F., Ector H., Stalpaert G., De Geest H. (1987). Ventricular tachycardia as a complication of annular subvalvular ventricular aneurysm in a Caucasian woman. *European Heart Journal*.

[16] Martinelli M., Rassi A., Marin-Neto J. A. (2013). CHronic use of amiodarone aGAinSt implantable cardioverter-defibrillator therapy for primary prevention of death in patients with Chagas cardiomyopathy Study: rationale and design of a randomized clinical trial. *American Heart Journal*.

